# Correlated X‐Ray and Electron Microscopies of a Single Biphasic GaAs Nanowire

**DOI:** 10.1002/smtd.202500740

**Published:** 2025-08-07

**Authors:** Thomas Dursap, Tao Zhou, Maxime Dupraz, Stéphane Labat, Olivier Thomas, Niels Fardeau, Philippe Regreny, Michel Gendry, Solène Brottet, Nicholas P. Blanchard, Martin V. Holt, Marie‐Ingrid Richard, Alexandru Danescu, José Penuelas, Matthieu Bugnet

**Affiliations:** ^1^ CNRS, ECL, INSA Lyon, UCBL CPE Lyon, INL, UMR 5270 69130 Ecully France; ^2^ Center for Nanoscale Materials Argonne National Laboratory Lemont IL 60439 USA; ^3^ Univ. Grenoble Alpes, CEA Grenoble IRIG, MEM NRX, 17 rue des Martyrs Grenoble 38000 France; ^4^ ESRF ‐ The European Synchrotron 71 Avenue des Martyrs Grenoble 38000 France; ^5^ Aix Marseille Université, CNRS Université de Toulon IM2NP UMR 7334 13397 Marseille France; ^6^ Université Claude Bernard Lyon 1, CNRS Institut Lumière Matière F‐69622 Villeurbanne France; ^7^ CNRS, INSA Lyon Université Claude Bernard Lyon 1 MATEIS, UMR 5510 69621 Villeurbanne France; ^8^ Present address: IMEC Kapeldreef 75 Leuven 3001 Belgium; ^9^ Present address: Université Claude Bernard Lyon 1, CNRS/IN2P3, IP2I Lyon UMR 5822 Villeurbanne 69622 France; ^10^ Present address: ANAXAM, Park Innovaare Parkstrasse 1 Villigen 5234 Switzerland

**Keywords:** bending, deformation mechanisms, III‐V semiconductors, nanowires, scanning X‐ray diffraction microscopy, twisting

## Abstract

Engineering the properties of semiconductors by changing their crystalline phase is a technologically and economically relevant alternative to doping using foreign elements, with strong potential for photonic and electronic applications. Although major advances have been reported recently for crystal‐phase engineering of III‐V and group IV semiconductor nanowires, interfacing two mismatched crystalline phases in a nanostructure induces several deformation mechanisms, which remain largely unexplored. Here, using state‐of‐the‐art synchrotron X‐ray nanobeam diffraction and transmission electron microscopy, subtle twisting and bending is unveiled within an individual GaAs nanowire containing cubic and hexagonal segments. Their role is discussed in accommodating the inter‐reticular spacing fluctuations, and their variations are correlated to the nanoscale phase distribution and to the effect of the NW support. This study brings direct evidence of a complex combination of deformation mechanisms in biphasic nanowires, which opens a new path to tune the nanowire properties with appealing perspectives for device engineering in nanophotonics and nanomechanics.

## Graphical abstract

Nano‐focused scanning X‐ray diffraction microscopy at a synchrotron light source, combined with transmission electron microscopy, reveals a clear correlation between the nanoscale distribution of cubic and hexagonal segments in a GaAs nanowire with subtle variations in inter‐reticular spacing, bending, and twisting. The interplay between deformation mechanisms and crystal structure, as well as the role of the NW support are discussed in the framework of linear elasticity.

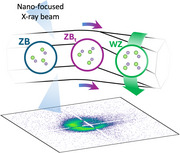

## 1 Introduction

The crystalline phase of a material is an intrinsic characteristic, which in the case of a semiconductor determines most of its physical properties, such as the bandgap and carrier mobility. Some semiconductors such as transition metal dichalcogenides and III‐V exist as different polymorphs at room temperature, therefore by controlling their crystalline phase it is possible to fabricate heterostructures for, e.g., photonic or electronic applications.^[^ ^1^, ^2^ ^]^ However, thus far, electronic devices mainly rely on controlling doping levels in semiconductors to tune their physical properties. This approach has been adopted for decades, and the current technologies have reached a high maturity level with limited perspectives of evolution.^[^ ^3^ ^]^ Engineering the properties of semiconductors by changing their crystalline phase without adding foreign elements appears to be an original, technologically and economically relevant solution to provide an alternative to doping and pave the way toward future electronics. Most group IV and III‐V semiconductors exhibit two different crystalline phases of cubic and hexagonal symmetry, namely zinc‐blende (ZB) and wurtzite (WZ), respectively.^[^ ^4^, ^5^ ^]^ However, making crystalline‐phase heterostructures in a controlled manner remains one of the biggest challenges to bridge the gap toward industrial applications. Major advances have been reported over the last few years for crystalline‐phase engineering of III‐V^[^ ^6^, ^7^, ^8^, ^9^ ^]^ cores and group IV shells^[^ ^10^, ^11^ ^]^ or branches^[^ ^12^ ^]^ in nanowire geometries, leading to, e.g., crystalline phase quantum dots of interest for quantum information technologies.^[^ ^13^ ^]^ When interfacing two different crystalline phases of the same compound in a single nanostructure, such as a nanowire (NW), several fundamental phenomena may occur as a result of the lattice mismatch between the WZ and ZB structures, and remain unclear, essentially because the concept of heteroepitaxy has been developed in the framework of thin films. Indeed, the free surfaces of NWs make them prone to various deformation mechanisms, which include strain but also tilting, bending, twisting, etc. Specifically, the recent observation of bending and twisting in semiconductor NWs^[^ ^14^ ^]^ suggests they could be considered as new degrees of freedom to tailor the electronic properties of NWs. However, the interplay between strain relaxation mechanisms in nanowires is intrinsically more complex than in planar heterostructures and is largely unexplored.

GaAs NWs containing WZ and ZB phases appear as an ideal system to determine the interplay between such phenomena. Strain variations in GaAs NWs containing WZ and ZB phases were evidenced by spatially resolved micro‐Raman spectroscopy, and were linked to the relative percentage of both phases along the nanowire.^[^ ^15^ ^]^ The strain induced by the WZ‐ZB phase change was shown to also affect the band alignment in GaAs^[^ ^16^ ^]^ and InAs^[^ ^17^ ^]^ nanowires. Moreover, the observation of heavy bending and twisting in strained core‐shell GaAs‐GaInP NWs^[^ ^18^ ^]^ suggests that such deformation mechanisms may also be present in NWs containing WZ and ZB structures of the same composition, e.g., GaAs. These additional pathways for strain relaxation have sparked considerable interest, however their interaction in NWs requires further investigation. Although strain has been evidenced at WZ‐ZB interfaces^[^ ^16^, ^19^ ^]^ and connected to the difference of lattice constant between the WZ and ZB structures,^[^ ^15^ ^]^ its quantitative analysis at the nanometer‐scale coupled with evidence of other deformation phenomena is lacking.

Whether induced by composition, crystalline phases, morphology, or overall microstructure, strain plays a significant role in NW properties. While sometimes undesired, lattice strain has been used to tune the properties of semiconductor NWs through strain engineering,^[^ ^20^, ^21^, ^22^, ^23^ ^]^ and foster their optical^[^ ^24^, ^25^ ^]^ and electronic^[^ ^14^, ^26^ ^]^ capabilities otherwise inaccessible from their relaxed lattices. A strong interplay between strain and lattice mismatch exists through relaxation mechanisms enabling epitaxy to be retained at interfaces, e.g., in InAs/InSb and GaAs/GaSb heterostructures,^[^ ^27^ ^]^ and in InGaN/GaN dot‐in‐a‐wire nanostructures^[^ ^28^ ^]^ subject to chemical ordering.^[^ ^29^ ^]^ The precise quantification of strain with nanometer‐scale resolution in a single nanoobject remains challenging and seldom achieved. Nevertheless, quantifying deformations accurately in low‐dimensional materials is increasingly relevant given the potential technological applications of nanomaterials, NWs in particular. The advent of state‐of‐the‐art X‐ray optics and next‐generation synchrotron sources has enabled the reliable quantitative evaluation and imaging of strain in nanoscale materials,^[^ ^30^, ^31^, ^32^, ^33^, ^34^ ^]^ up to three dimensions^[^ ^35^, ^36^ ^]^ and during (electro)catalytic reactions^[^ ^37^, ^38^ ^]^ using coherent diffraction imaging. Using the capabilities of scanning X‐ray diffraction microscopy (SXDM) at a synchrotron facility enables mapping strain fields over μm‐size areas with spatial resolution of a few tens of nm^[^ ^31^ ^]^ and obtaining an excellent precision on the strain values unmatched by any other techniques. SXDM was used recently to evaluate the interplay between structure and strain in single GaAs/In_0.2_Ga_0.8_As/GaAs core–shell NWs,^[^ ^39^ ^]^ and it allowed nanoscale mapping of the full strain tensor, rotation, and composition in partially relaxed In_*x*_Ga_1 − *x*_N layers.^[^ ^40^ ^]^ The same approach was used to explore the strain distribution and bending inhomogeneity in strongly bent core–shell NWs with asymmetric shells in the (In,Ga,Al)As system,^[^ ^41^ ^]^ as well as the effect of Joule heating during resistivity measurements in individual GaAs NWs on their structure, tilt and bending.^[^ ^42^ ^]^

**Figure 1:**
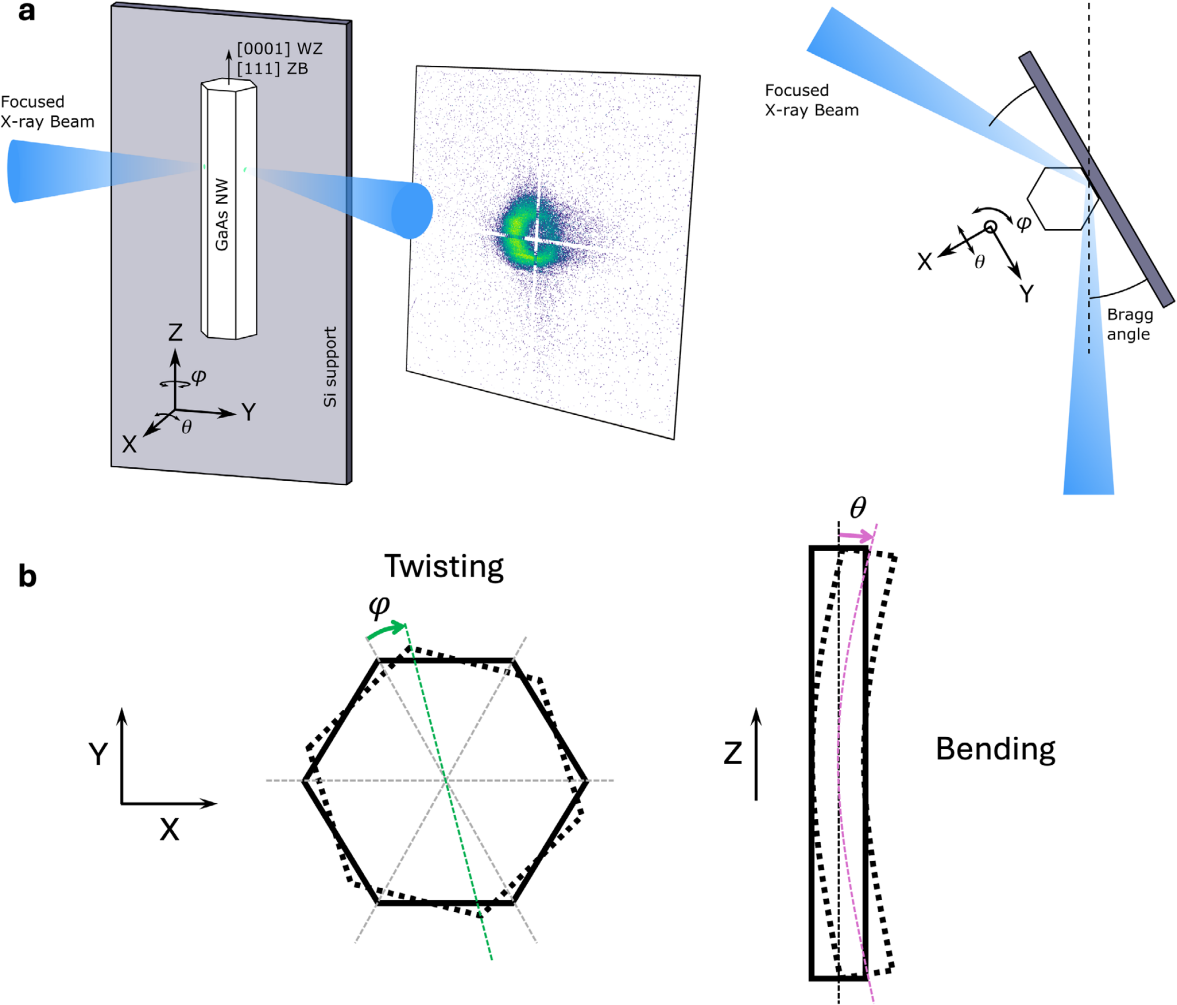
Scanning X‐ray diffraction microscopy to probe strain, bending and twisting of a nanowire. a) Schematic showing the side view and top view orientation of the system. The azimuthal angle *φ*, corresponding to the torsion along the long axis of the NW, and the tilt / orientation angle *θ* representing the bending of the NW of the (hkl) planes are represented regarding the NW orientation. b) Illustration of twisting and bending in a NW. The thick black lines denote the position of the NW in relaxed (full) and bent/twisted (dashed) configuration. The thin dashed lines are a guide to highlight the twist angle (green arrow) and bend angle (purple arrow).

Here, we provide evidence of the relaxation mechanisms at play within a single GaAs nanowire containing a random sequence of cubic (ZB and twinned ZB (ZB_*t*_)) and hexagonal (WZ) crystalline phases, by combining state‐of‐the‐art synchrotron radiation SXDM, as illustrated in **Figure** **1**a, and (scanning) transmission electron microscopy ((S)TEM). The excellent complementarity of both techniques shows how the lattice mismatch between the two phases induces a deformation along the nanowire axis, and a clear correlation with the nanowire bending and twisting, which could be attributed to an original relaxation mechanism. The results, supported by linear elasticity calculations, demonstrate the complex effect of the ZB‐WZ phase transition on the inter‐reticular spacing of both ZB and WZ phases. Notably, the SXDM experiments provide clear evidence of subtle twisting and bending of the NW, as illustrated in Figure 1b, which correlate remarkably well with the localization of the ZB_*t*_ and WZ segments, respectively. Finally, the effect of the contact between the NW and its support on the deformation mechanisms is discussed.

## 2 Results and Discussion

### 2.1 Phase Distribution

**Figure 2:**
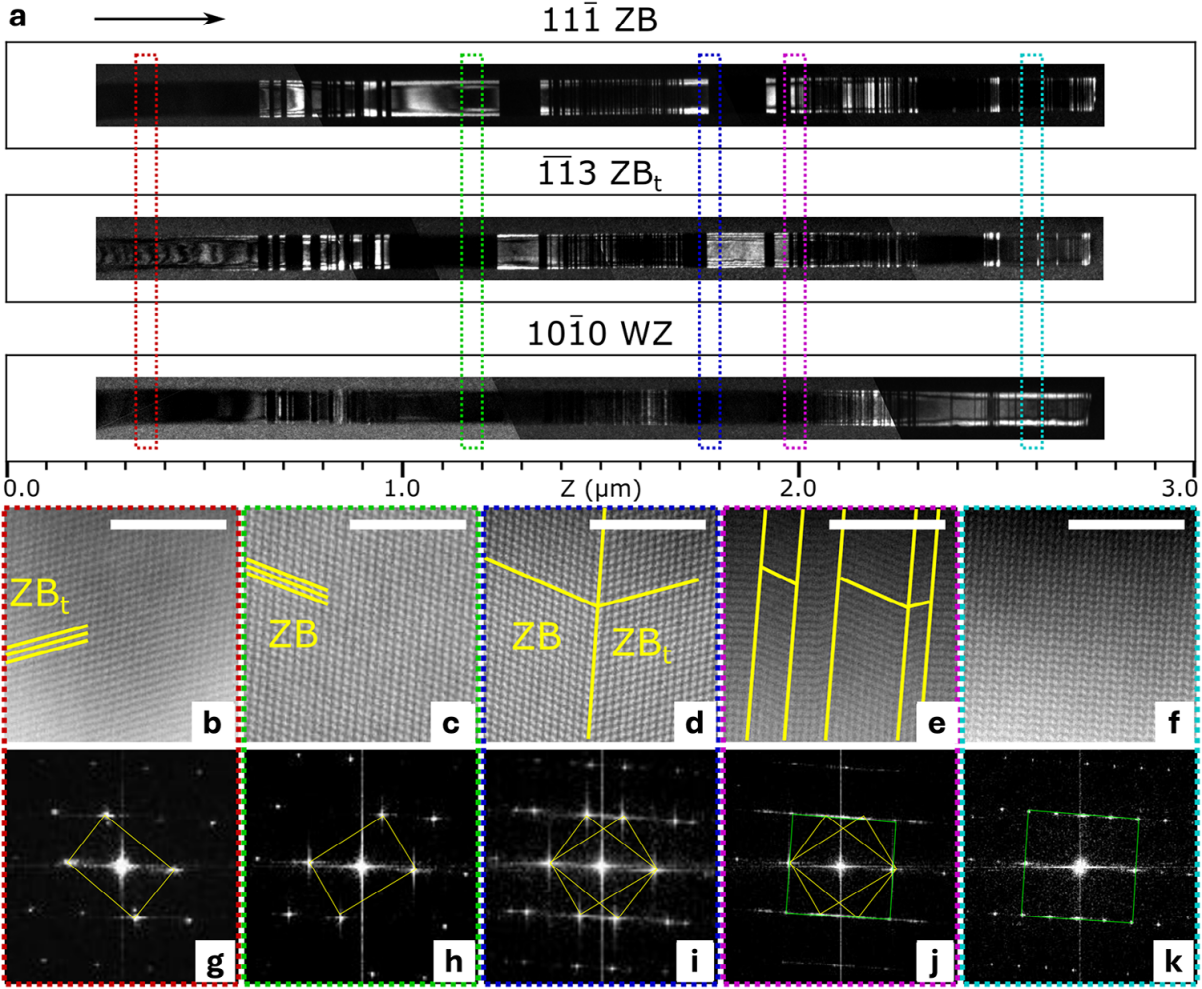
Phase distribution within a GaAs nanowire from dark field TEM and HAADF STEM. a) DF‐TEM imaging of the ZB, ZB_*t*_ and WZ in a nanowire. The arrow indicates the growth direction. b–f) High‐resolution HAADF STEM images along the [1$\bar{1}$0] zone axis showing the crystal structure at specific position highlighted by the colored dashed rectangles in (a). Scale bars are 5 nm. The overimposed thick yellow lines are a guide for the eye to help identifying the ZB and ZB_*t*_ structure. g–k) FFTs associated to the HR‐STEM images in (b–f), respectively. (g,h) and (k) FFTs show the typical diffraction pattern of the ZB_*t*_, ZB and WZ structures, respectively. The thin yellow and green lines in the FFT patterns are a guide for the eye to help identifying the pattern of each structure.

**Figure 3:**
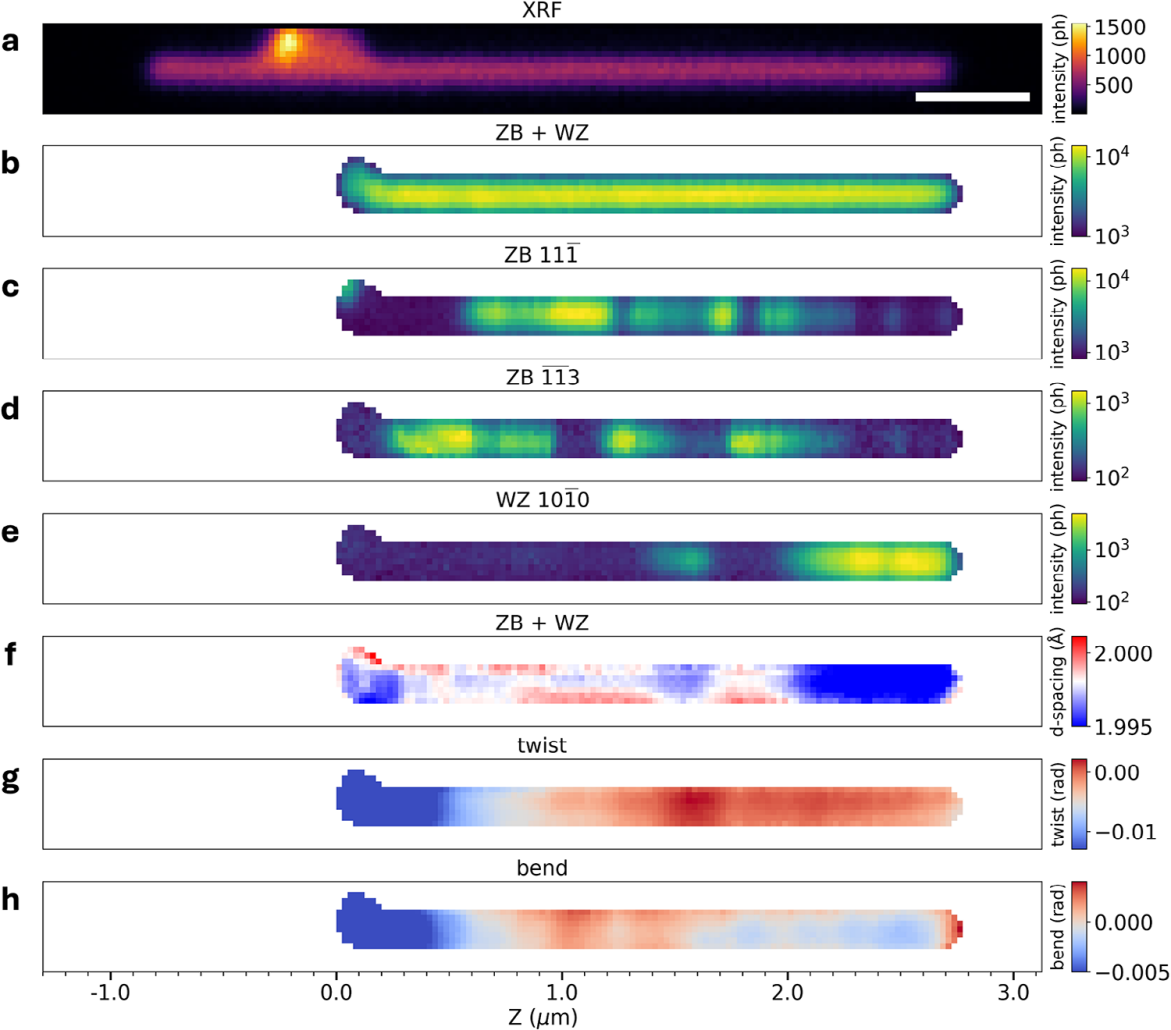
Inter‐reticular spacing, twisting and bending from scanning X‐ray diffraction microscopy. a) X‐ray fluorescence map, scale bar is 500 nm, b–e) diffraction intensity maps of ZB(20$\bar{2}$)/WZ(2$\bar{1}$ $\bar{1}$0) common diffraction spot (b), ZB(11$\bar{1}$) (c), ${\mathrm{ZB}}_{t}(\bar{1}$ $\bar{1}$3) (d), and WZ(10$\bar{1}$0) (e); f) *d*‐spacing map extracted from the ZB(20$\bar{2}$)/WZ(2$\bar{1}$ $\bar{1}$0) common diffraction spot, g) twist angle (*φ*) map, and h) bend angle (*θ*) map.

The NW studied by nano‐focused SXDM was extracted into a thin lamella for dark field (DF)‐TEM and STEM analysis, using the focused ion beam (FIB) lift‐out preparation approach (see Methods), thus enabling a correlative study on a single NW. The NW of interest is ∼3.5‐μm long and ∼90 nm in diameter, as shown in Figure S1 (Supporting Information). To obtain a complete view of the phases and their distribution over the full length of the NW, DF‐TEM was performed. The NW contains three phases: cubic ZB and its twinned structure ZB_*t*_, and hexagonal WZ. By selecting similar reflections as used in the SXDM experiments, DF‐TEM images of the ZB, ZB_*t*_ and WZ segments are illustrated in **Figure** **2**a. These DF‐TEM images reveal the localization of the pure phase segments and the mixed phase portions with sub‐nm spatial resolution, better than the SXDM maps in **Figure** **3**, where a resolution of ∼25 nm has been achieved. As visible in Figure 2a, the ZB and ZB_*t*_ phases appear successively in segments of various lengths ranging from few nms to few hundreds of nm. The WZ phase appears in two separate segments, at position Z ∼1.55 μm in the middle of the NW and for the last ∼700 nm of growth. Aberration‐corrected high angle annular dark field (HAADF) STEM enables an atomic‐scale view of the NW structure at specific locations, as shown in Figure 2b–f. These observations, along with the corresponding fast Fourier transforms (FFTs) in Figure 2g–k, confirm the presence of pure WZ, ZB, ZB_*t*_, mixed ZB and ZB_*t*_, and mixed WZ, ZB and ZB_*t*_ at specific locations of the NW. Structural transitions leading to ZB_*t*_ phases typically occur when a cubic‐hexagonal phase change is initiated from the growth parameters.^[^ ^9^ ^]^ Although HAADF STEM provides high resolution information down to the sub‐Å scale, its limited field of view inherently restricts the investigation to a few selected parts of NW segments. Therefore, complementary SXDM analyses with a spatial resolution of ∼25 nm performed on the entire NW are discussed in the following.

The NW was studied by SXDM at the hard X‐ray nanoprobe beamline 26‐ID of the Advanced Photon Source (APS) by using a nano‐focused beam with a diameter of ∼25 nm. Figure 3a shows the X‐ray fluorescence (XRF) image of the NW located using the Ga K_α_ signal. The bright spot visible at the top left, near the base of the NW, corresponds to a fragment of another NW, which can also be seen in Figure S1 (Supporting Information). For all SXDM measurements, scans were systematically stopped before reaching the base of the NW to avoid diffraction from the small fragment. Figure 3b shows a DF image of the NW, obtained by integrating the intensity from the ZB(20$\bar{2}$) and WZ(2$\bar{1}$ $\bar{1}$0) reflections. These reflections are common to all WZ, ZB and ZB_*t*_ phases, as confirmed by the uniform integrated intensity value throughout the entire NW segment in Figure 3b. The distributions of ZB, ZB_*t*_ and WZ structures within the NW were obtained using the integrated intensity from the ZB(11$\bar{1}$), ${\mathrm{ZB}}_{t}(\bar{1}$ $\bar{1}$3) and WZ(10$\bar{1}$0) Bragg reflections, respectively, as shown in Figure 3c–e. Despite the spatial resolution of SXDM being limited to ∼25 nm, the phase distribution in SXDM maps correlates remarkably well with that of DF‐TEM images in Figure 2.

### 2.2 Interplay Between Inter‐Reticular Spacing, Twisting and Bending

To extract information about the inter‐reticular spacing (*d*‐spacing) of the planes normal to the growth direction, twisting and bending, it is necessary to measure the complete 3D reciprocal space around a selected Bragg reflection. With SXDM this is achieved by repeating the 2D spatial maps at slightly different incident angles around the selected Bragg reflection in a procedure known as tilt‐series.^[^ ^31^ ^]^ The *d*‐spacing, twisting (*φ*) and bending (*θ*) derived from the common ZB(20$\bar{2}$) and WZ(2$\bar{1}$ $\bar{1}$0) reflections are shown in Figure 3f‐h, respectively. These reflections, common to ZB, ZB_*t*_ and WZ, are specifically chosen here to allow the probing of the entire NW at once. The extracted *d*‐spacing shown in Figure 3f reveals distinct regions along the NW. The regions with small *d*‐spacing (blue) match well the presence of WZ segments as indicated in Figure 3e, while regions with large *d*‐spacing (red) correspond to the phase distribution visible in the maps built from the ZB(11$\bar{1}$) (Figure 3c) and ${\mathrm{ZB}}_{t}(\bar{1}$ $\bar{1}$3) (Figure 3d).

**Figure 4:**
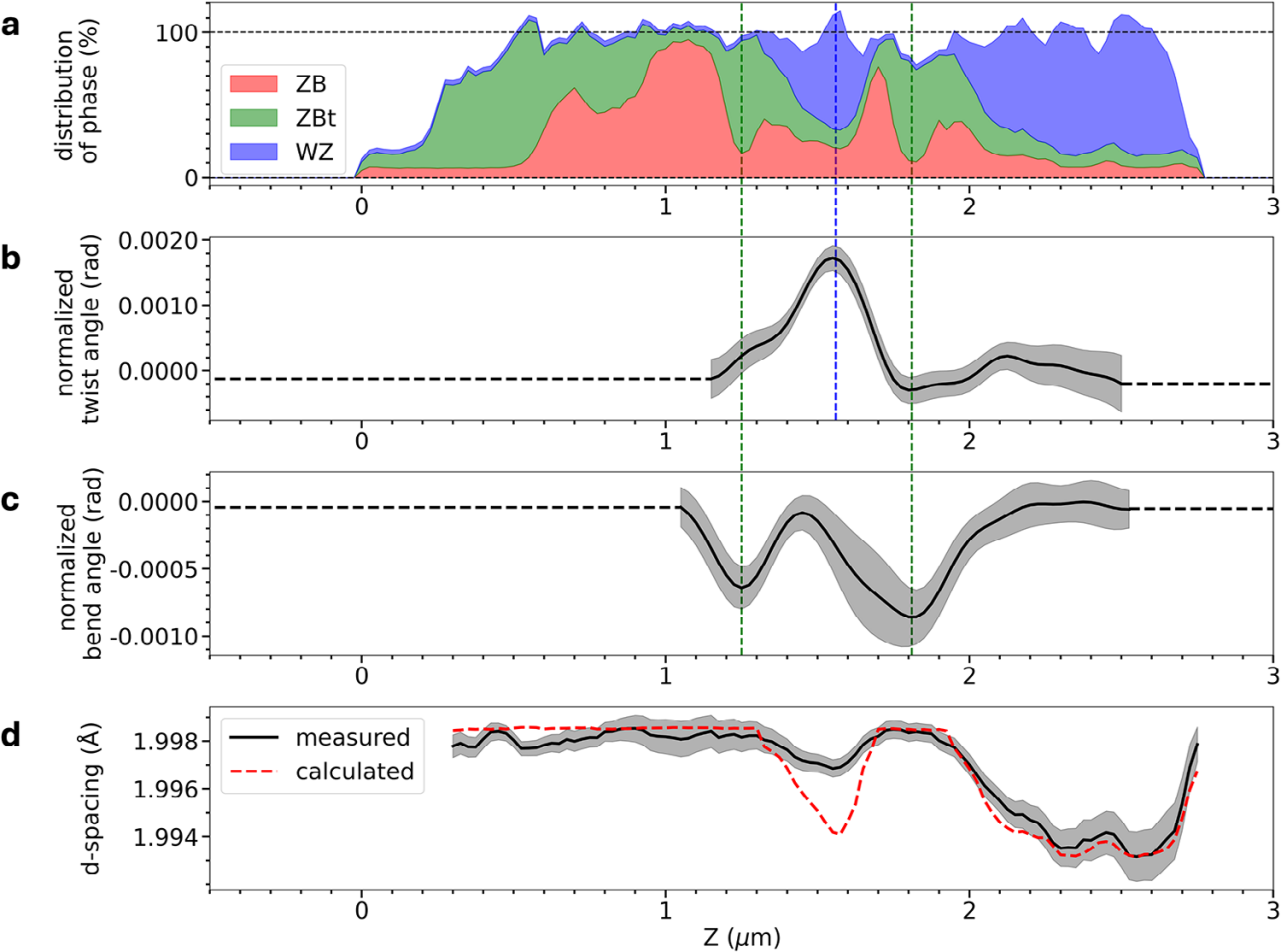
Correlation of crystal structure with deformation mechanisms. a) Normalized intensity of ZB(11$\bar{1}$), ${\mathrm{ZB}}_{t}(\bar{1}$ $\bar{1}$3), and WZ(10$\bar{1}$0) diffraction maps shown in Figure 3, as a function of position along the NW. The intensity of each phase is proportional to its area under the curve. b) Evolution of the twist angle *φ* along the NW length, c) bend angle *θ*, and d) *d*‐spacing. b–d) are line profiles of the 2D‐maps shown in Figure 3. The grey area indicates the error bar, see Methods.

**Figure** **4**a shows a distribution of phase in the NW, calculated using normalized intensity profiles of the ZB(11$\bar{1}$), ${\mathrm{ZB}}_{t}(\bar{1}$ $\bar{1}$3), and WZ(10$\bar{1}$0) Bragg reflections, respectively. After normalization to the maximum value of each phase distribution, the sum of all three phases adds up to ∼100 ±10% except near Z = 0 μm, where large rotation induced by the broken NW fragment shifts the Bragg peaks out of the angular range of the measurements. Based on this distribution of phase, we have calculated a theoretical *d*‐spacing using a linear combination of experimental values from the literature ($d{\left(20\bar{2}\right)}_{ZB}$ = 1.99874 Å,^[^ ^43^ ^]^ $d{(20\bar{1}\bar{1})}_{\mathrm{WZ}}$ = 1.99225 Å^[^ ^44^ ^]^) weighed by the proportion of each phase in the area illuminated by the beam (see Methods). A comparison was made with the experimentally determined *d*‐spacing from Figure 3f. The result of the comparison is shown in Figure 4d. The experimentally determined *d*‐spacing is ∼1.998 Å in the ZB and ZB_*t*_ regions, while it decreases down to 1.997 Å where a ∼200‐nm‐long WZ segment is located at position Z ∼1.55 μm in the middle of the NW. Toward the top of the NW, where a ∼700‐nm‐long WZ segment is present, the *d*‐spacing drops below 1.994 Å. The qualitative variations of the *d*‐spacing are in agreement with the distribution of both WZ and ZB/ZB_*t*_ structures, which matches the growth conditions used during the fabrication of this sample, as described in Methods. The calculated *d*‐spacing (red dashed line in Figure 4d) roughly matches the experimental values in the ZB regions at Z ⩽1.3 μm and Z ∼1.8 μm, as well as in the long WZ segment at Z ⩾2.3 μm. Notable discrepancies remain however, quite visibly in the short WZ segment at Z ∼1.55 μm, and in a less pronounced manner at the beginning of the long WZ segment at Z ∼2.1‐2.2 μm.

The global trend and subtle variations in the experimentally measured inter‐reticular distances were also reproduced by a linear elasticity model applied to the WZ segment between ∼2.25 μm and ∼2.7 μm (see Figure S4, Supporting Information). The stacking faults, visible as dark thin lines across the diameter of the NW in the DF‐TEM WZ image in Figure 2a, correspond to small inclusions of ZB and ZB_*t*_ structures and account for the *d*‐spacing variations observed in SXDM.

**Figure 5:**
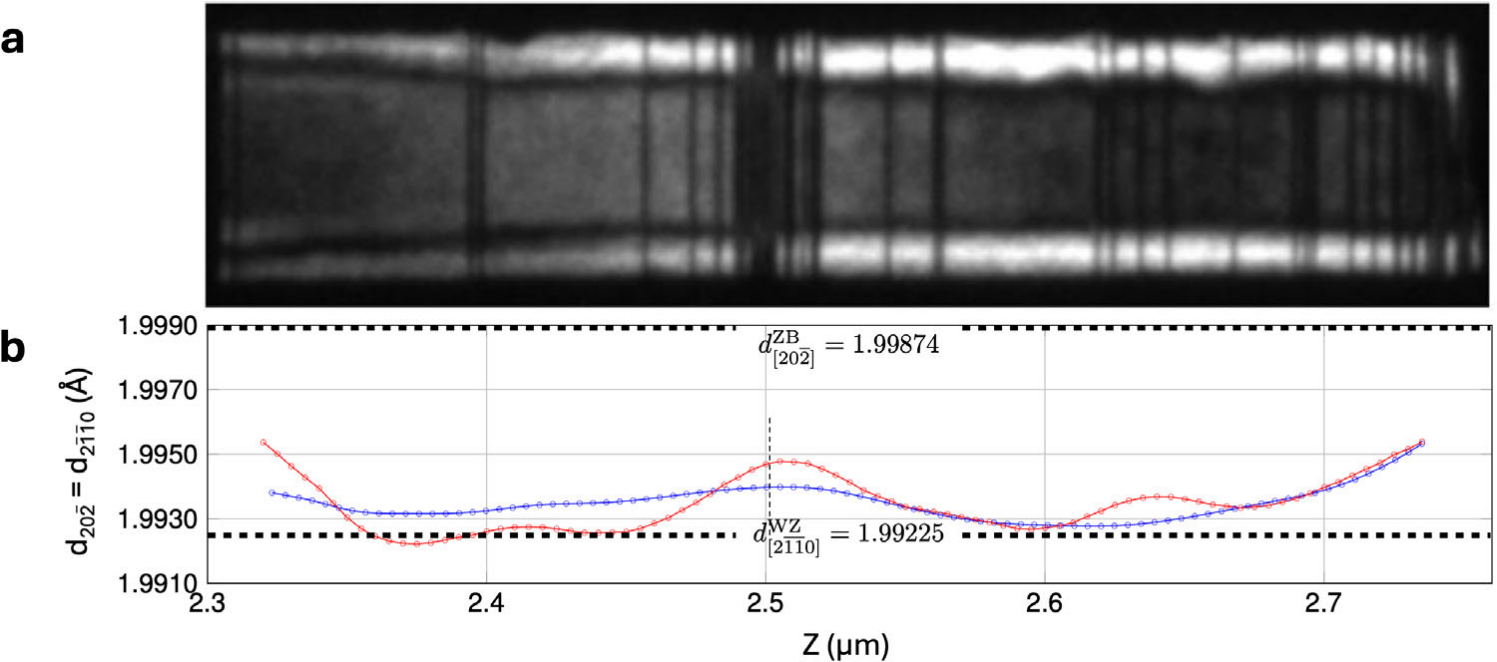
Linear elasticity model for *d*‐spacing. a) DF‐TEM image along [10$\bar{1}$0] of the main WZ segment near the head of the NW in the range Z ∼2.3–2.75 μm of Figure 2. b) Inter‐reticular distance ZB $d_{\left(20\bar{2}\right)}$/ WZ $d_{\left(2\bar{1}\bar{1}0\right)}$ measured (blue line) and calculated (red line) for an average NW diameter of 50 nm. Top and bottom dashed lines represent the theoretical values of the ZB $d_{\left(20\bar{2}\right)}$ and WZ $d_{\left(2\bar{1}\bar{1}0\right)}$ inter‐reticular distances, respectively, as extracted from the literature.^[^ ^43^, ^44^ ^]^

The top ∼450 nm of the NW of the DF‐TEM WZ image obtained by selecting the WZ(10$\bar{1}$0) reflection was segmented to identify the phase location (see Supporting Information), and to calculate the variation of *d*‐spacing (**Figure** **5**) along the NW axis. To determine the *d*‐spacing, the lattice parameters *a* and *c* have to be calculated (see Supporting Information). In the pure‐phase segments and for the portions away from the phase transitions, the lattice parameters were approximated to their theoretical values. However, due to the difference in the lattice parameters between ZB and WZ, lattice strain is expected close to the areas where stacking faults and structural changes occur, thus slightly modifying the values of the lattice parameters to accommodate these variations.

The linear elasticity model supports the observation of local maxima of *d*‐spacing values in the experimental map in Figures 3f and 4d. The calculated values of *d*‐spacing along the NW are reported in Figure 5b (red line), where they are compared to the experimental measurements of the *d*‐spacing (blue line). From ∼2.32 μm to 2.37 μm, both experimental and calculated *d*‐spacing decrease, as expected from the onset of the WZ structure with respect to ZB and ZB_*t*_, as shown in Figure 2a. A local maximum of the inter‐reticular distance is observed just above ∼2.4 μm in both the experimental and simulated curves in Figure 5b, which appears to be correlated to stacking faults. An intense and broad maximum is observed just above ∼2.5 μm in both experimental and calculated curves, which is linked to the high density of stacking faults and multiple phase changes visible in Figures 2a and 3c–e. At ∼2.63 μm, the calculated *d*‐spacing hits a local maximum, which is not visible in the experiment, and which corresponds to the high density of stacking faults. Above ∼2.67 μm, the calculated *d*‐spacing increases consistently and overlaps with the experimental value. Overall, the experimental results appear less sensitive to the phase changes compared to the simulations, which is very likely due to the lower resolution of the experiment and the simplicity of the model, like the non‐discrimination between ZB and ZB_t_. The fact that the experimental *d*‐spacing appears insensitive to the presence of a high density of stacking faults, e.g., at ∼2.63 μm, whereas it is affected by a low density of stacking faults, e.g., just above ∼2.4 and ∼2.5 μm, suggests the presence of additional defects that are not considered in the model. Nevertheless, the global trend and the main variations of experimentally measured inter‐reticular distance are reproduced by the linear‐elasticity model. These results highlight the strong contribution of structural transitions on the *d*‐spacing.

To rationalize these observations, the SXDM data were used to investigate further deformation mechanisms of the NW. Specifically, twisting and bending can be extracted from the 3D Bragg peak positions (see Methods). As such the magnitudes of twisting and bending are calculated as a function of position along the NW, and represented as 2D maps in Figure 3g,h. The experimental data show that the NW is twisted up to nearly ∼1.7 mrad in the middle of the NW (Figure 3g), which corresponds to the position of the WZ segment centered at Z ∼1.55 μm. It also appears that the NW displays an inhomogeneous bending distribution from ∼1 to 2.5 μm (Figure 3h). The correlation of twist (*φ*) and bend (*θ*) angles with the phase distribution and *d*‐spacing along the NW is summarized in Figure 4. In this representation, the correspondence between the maximum twist value of ∼1.7 mrad and the position of the WZ segment in the middle of the NW is clear. Specifically, the twisting contribution (Figure 4b) becomes significant where the calculated *d*‐spacing is notably lower than the experimental value, at Z ∼1.55 μm, as shown in Figure 4d. In a more subtle manner, the local twisting maximum centered at Z ∼2.1 μm also corresponds to a position in the top WZ segment where the measured *d*‐spacing is higher than calculated. Therefore, it appears that twisting may compensate longitudinal strain along the NW axis, visible through *d*‐spacing variations, in WZ regions up to few hundreds of nm in length following epitaxy on the ZB structure. The stronger twist at Z ∼1.55 μm might also arise from the fact that the corresponding WZ segment is not pure as it actually contains short sections of ZB and ZB_*t*_ (see Figures 2 and 4a), and that this segment is sandwiched between two ZB sections, thus forcing the lattice to maintain some twist. This is different to the WZ segment at Z ⩾ 2 μm, for which the twist amplitude is much lower due to relaxation facilitated by the free NW tip.

The NW is also bent with varied amplitude along its length. The maxima of the bend angle, located at ∼1.25 μm (∼0.6 mrad) and ∼1.8 μm (∼0.8 mrad) as shown in Figure 4c, match the positions along the NW where ZB_*t*_ is the most abundant phase (Figure 4a). This striking correspondence suggests that the structural transition from ZB (peaked at ∼1.1 μm and ∼1.7 μm) to ZB_*t*_ (peaked at ∼1.25 and ∼1.8 μm) induces subtle bending of the NW up to ∼0.9 mrad. Although this absolute value of bending is rather small, it is unmistakably measurable beyond the margin of error along the NW using SXDM. The SXDM spatial resolution proves sufficient to detect and map twisting and bending in long segments of WZ and ZB, respectively, while the effect of stacking faults localized by DF‐TEM appears limited on twisting, bending and d‐spacing.

Several hypotheses can be expressed to explain the correspondence between twisting and WZ, and between bending and ZB_*t*_. Changes in the width of the NW facets induced by the ZB to WZ phase transition could lead to twisting via surface stress, as suggested theoretically.^[^ ^45^ ^]^ The ZB to ZB_*t*_ transition, however, is not expected to introduce such structural modifications. This recent theoretical work has shown that twisting induces a subtle displacement field along the NW axis, suggesting that the structural implications of twisting on bending (and vice‐versa) should also be explored to understand some of the experimental observations reported here. It is also worth noting that surface elasticity can play an important role on the deformation of NWs, as shown theoretically.^[^ ^45^, ^46^ ^]^ In these works, it has been demonstrated that the anisotropy in surface stress can cause warping in the NW. Such phenomenon has been observed experimentally in Au NWs.^[^ ^47^ ^]^ Secondly, the intrinsic crystallographic differences of hexagonal and cubic structures may explain why WZ segments tend to exhibit twisting behavior, while ZB segments are more prone to bending. In WZ structures, the bonds in the hexagonal lattice are less constrained along the axis of twisting, making it easier for the nanowire to undergo torsional deformation. This phenomenon has been evidenced in hexagonal SiC and was explained by basal plane dislocations inducing plastic deformation.^[^ ^48^ ^]^ Conversely, complex shear stresses applied to face‐centered‐cubic lattices eventually lead to twisting.^[^ ^49^ ^]^

Finally, the influence of the substrate on the complex deformation mechanisms of the NW could be evaluated. By comparing coherent diffraction patterns from suspended Au NWs, Z. Ren^[^ ^50^ ^]^ clearly evidenced a strong influence of the Si support. The diffraction patterns of the suspended parts showed perfect patterns, while those of the contact parts showed complex features that could be explained by bending. A similar behavior cannot be excluded here, where the contact forces between the GaAs NW and its support may induce bending. However, although the reason for preferential bending where the cubic phase is located, in contrast to the hexagonal phase, remains to be determined, the influence of the support on the full state of deformation of the GaAs NW, which includes the twist, is of interest to understand the coupling between the deformation mechanisms. It should be emphasized that the twist values reported in Figure 4b must be interpreted with care. The WZ lattice at Z ∼1.55 μm is misoriented by up to 1.7 mrad with respect to the ZB lattice, which can be interpreted in two ways. Indeed, the plot suggests that the twist could be either achieved within the WZ segment and reach its maximum at Z ∼1.55 μm, or occur within the ZB lattice at Z < 1.3 μm (i.e. prior to the WZ growth) and decrease within the WZ down to a minimum value at Z ∼1.55 μm. A recent theoretical investigation^[^ ^51^ ^]^ of GaAs@InAlAs NWs, stressed on one side, shows that a coupling between twist and bending may occur in the ZB phase in (vertical) non‐symmetric geometries. Since the GaAs NW in the present work is deposited on a support, it is reasonable to assume that the interaction of one of the ZB $\left(\bar{1}10\right)$ facets with the support provides elastic energy to induce a twist. Indeed, when the NW is subjected to bending within the (Y, Z) plane, as defined in Figure 1, twisting of the NW cannot be induced by bending alone. In order to check the potential effect of the support, a 3D numerical simulation was performed on a 0.9‐µm‐long NW segment made of a ZB(0.3 μm)/WZ(0.4 $\mu m){/\mathrm{ZB}}_{t}$(0.2 μm) stacking sequence (see Figure S5, Supporting Information), thus mimicking the studied NW in the range 1.1–2 μm (Figure 4). The NW is assumed to lay flat on a $\left(\bar{1}10\right)$ facet at a constant X value. The value of the twist angle *φ* as a function of the axial coordinate is illustrated in Figure S6 (Supporting Information). Qualitatively, the profile obtained is consistent with the measured values presented in Figure 4 as the twist angle reaches its maximum value in the middle segment (WZ). This is due to the cumulative nature of the twist, and the ZB and ZB_*t*_ have opposite effects on the twist. The latter is the consequence of the fact that the elastic coupling coefficients (in Voigt notation *C*_14_, *C*_24_ and *C*_56_) change sign from ZB to ZB_*t*_. One notes, however, that despite its consistent evolution along the 0.9‐µm‐long ZB/WZ/ZB_*t*_ NW model the theoretical twist values are one order of magnitude smaller than the experimental ones. This apparent mismatch could originate from limitations of the theoretical framework, the assumption that the ZB $\left(\bar{1}10\right)$ facet is fully in contact with the support, as well as the rather simplistic model of the NW (see Figure S5, Supporting Information) whereas it does not contain pure ZB, WZ, and ZB_*t*_ phases over hundreds of nanometers as evidenced in Figure 4a. This discrepancy also suggests that even though the support plays a role in promoting a twist of the NW, other sources of the twist may be necessary to explain its magnitude.

## 3 Conclusion

In summary, the influence of structural changes on the deformation mechanisms in a GaAs NW containing ZB and WZ segments has been evaluated using correlated advanced characterization methods, including DF‐TEM, STEM and nano‐focused SXDM. The latter revealed the combination of subtle variations in inter‐reticular spacing, bending, and twisting along the NW length and enabled their quantification on the nanometer scale. This work also provides clear experimental evidence of the correlation between the nanoscale distribution of ZB, ZB_*t*_, and WZ, with the variations in inter‐reticular spacing, bending, and twisting. Specifically, the results suggest that twisting may compensate longitudinal strain along the NW axis in WZ regions up to few hundreds of nanometers in length, and that the structural transition from ZB to ZB_*t*_ induces subtle bending of the NW in the sub‐mrad range. Numerical simulations show that the twist seems to be driven, at least partially, by the interaction between the ZB segments and the support. The latter is thought to play an important role in the deformation mechanisms of nano‐objects like nanowires. These results open relevant and concrete perspectives in the field of nanophotonics and nanomechanics by engineering minute local deformations within single nanoobjects via a controlled crystal structure on the nanometer scale.

## 4 Experimental Section

### Nanowire Synthesis

The GaAs NWs were grown on epi‐ready (111) silicon substrates using a Riber R32 solid‐source MBE reactor. The substrate was cleaned in ultrasonic bath for 5 min in both acetone and ethanol and degassed in ultra‐high vacuum at 200°C before its introduction in the MBE reactor. The native SiO_2_ oxide layer present on the epi‐ready substrate was preserved to enable the self‐assisted growth.^[^ ^52^ ^]^ Catalyst Ga droplets were formed on the substrate by pre‐depositing Ga for 2 s at 450°C, with a deposition rate of 0.5 ML/s.^[^ ^53^, ^54^, ^55^ ^]^ This rate is quoted in units of equivalent growth rates of GaAs 2D layers, measured by reflection high energy electron diffraction (RHEED) oscillation on a GaAs substrate.^[^ ^56^ ^]^ The substrate was then heated at a growth temperature of 600°C. The growth of the NWs was finally initiated with the opening of the Ga and As fluxes. The Ga and As_4_ fluxes, valves and shutters were finely controlled during the growth using a homemade software, handling the MBE system. The NWs were grown with a Ga and As_4_ flux of 0.5 and 1.2 ML/s, respectively, corresponding to a V/III flux ratio of 2.4. The WZ part of the NWs was synthesized by changing the As_4_ flux from 1.7 to 2.6 ML/s, leading to a V/III flux ratio ranging from 3.3 to 5.2. The entire growth of the WZ section was monitored in real‐time using a RHEED system at 30 keV, allowing the in situ control of the crystal structure growth. The sample was rotating during the entire growth of the ZB section. The rotation was then stopped during the growth of the WZ segment, to allow real‐time RHEED control of the growth. Further description of the growth of WZ segments is available in references.^[^ ^9^, ^57^ ^]^

### Scanning X‐Ray Diffraction Microscopy—Data Acquisition

The NW was studied at the hard X‐ray nanoprobe beamline 26‐ID‐C of the Advanced Photon Source (APS) at Argonne National Laboratory, USA. The NWs were drop‐cast onto a 10‐μm‐thick silicon support transparent to hard X‐rays and prepared for this application via selective etching and lithography by Norcada Inc. The investigated NW had its *a*‐plane (2$\bar{1}$ $\bar{1}$0) facet parallel to the Si support. A photon energy of 10.4 keV was used to excite the Ga K_α_ signal at 10.367 keV. The emitted Ga fluorescence signal was used to align the diffraction maps acquired at different incident angles. The beam was focused down to a 25‐nm probe using a Fresnel zone plate. The diffraction signals were collected on a Medipix3 detector with 516×516 pixels^2^ and 55 µm pixel size, at a sample‐to‐detector distance of 0.9 m. The spatial step size for the diffraction mapping was ∼20 nm and the angular step size for the tilt‐series was 0.2 degree.

### Scanning X‐Ray Diffraction Microscopy—Data Processing

For each scanned sample point, its corresponding peak position was extracted by finding the intensity center of mass in the 3D reciprocal space. A change in the magnitude of the momentum transfer **Q** corresponds to a change in *d*‐spacing, with $d=\frac{2\pi }{\left|Q\right|}$. A change in the direction of the momentum transfer corresponds to a rigid rotation of the lattice. Using the definition illustrated in Figure 1a, the **Z** axis was chosen to be along the growth direction of the NW and the **X** axis was parallel to the surface normal of the Si support. The **Y** axis was orthogonal to both **Z** and **X**. The twist angle *φ* was defined as a rotation of the lattice around **Z** whereas the bend angle *θ* was defined as a rotation of the lattice around **X**.

For Figure 4a, the percentage of the different phases at each point of the NW was calculated by normalizing the integrated intensities of ZB, ZB_*t*_ and WZ with their respective maximum value. The error ±10% corresponds to the standard deviation of the sum of all phases. The broken NW segment near Z = 0 μm introduced a huge twist and tilt gradient as can be observed in Figure 3g,h. The *d*‐spacing in Figure 3f was unaffected as a rotation of the lattice does not change the magnitude of the momentum transfer. To extract the line profile shown in Figure 4b, we first averaged the twist angle value of the center five pixels in the radial direction of the NW as shown in Figure 3g. A spline background was fitted and subtracted which corresponded to the twist gradient induced by physical contact with the broken NW segment. To extract the line profile shown in Figure 4c, we first averaged the bend angle value of the center five pixels in the radial direction of the NW as shown in Figure 3h. For both twisting and bending profiles, the error bar was defined as the standard deviation within the center five pixels in the radial direction of the NW. A linear background was fitted and subtracted which corresponds to the twist gradient induced by physical contact with the broken NW segment.

### Electron Microscopy Sample Preparation and Experiments

The density and length of NWs was checked by scanning electron microscopy using the secondary electron detector of a JEOL 7401F SEM at an acceleration voltage of 10 kV. Scanning transmission electron microscopy (STEM) in high angle annular dark‐field (HAADF) imaging mode was performed using a JEOL JEM‐ARM200F NeoARM, equipped with a high brightness cold‐FEG and a CEOS ASCOR aberration‐corrector of the condenser lenses. Dark‐field (DF) TEM imaging and selected area electron diffraction were carried out in a JEOL JEM2100 equipped with a LaB_6_ gun. The transmission electron microscopes were operated at 200 kV. The DF image is obtained using only the electrons diffracted by specific crystallographic planes of the phases present in the NW. The TEM lamellas were prepared using conventional focused ion beam (FIB) milling, on an individual nanowire previously investigated by scanning X‐ray diffraction microscopy at the Advanced Photon Source (APS), the Argonne National Laboratory (ANL, USA) and precisely localized on the Si support.

## Conflict of Interest

The authors declare no conflict of interest.

## Supporting information

Supporting Information

## Data Availability

The data that support the findings of this study are available from the corresponding author upon reasonable request.
