# Supplementary material for: Correlated X‐Ray and Electron Microscopies of a Single Biphasic GaAs Nanowire
Source: Small Methods. 2025 Aug 7;10(3):2500740. doi: 10.1002/smtd.202500740 (PMC12893255; doi:10.1002/smtd.202500740)
Supplement: Supplementary file 1 — Supporting Information [file SMTD-10-2500740-s001.pdf]

# Supporting Information for Correlated X-ray and Electron Microscopies of a Single Biphasic GaAs Nanowire

T. Dursap,[1,2,*] T. Zhou,[3,*] M. Dupraz,[4,5,6] S. Labat,[7] O. Thomas,[7] N. Fardeau,[1,8] P. Regreny,[1] M. Gendry,[1] S. Brottet,[1] N. Blanchard,[9] M. V. Holt,[3] M.I. Richard,[10,5] A. Danescu,[1] J. Penuelas,[1,†] and M. Bugnet[11,‡]

[1] *CNRS, ECL, INSA Lyon, UCBL, CPE Lyon, INL, UMR 5270, 69130 Ecully, France*
[2] *Present address: IMEC, Kapeldreef 75, 3001 Leuven, Belgium*
[3] *Center for Nanoscale Materials, Argonne National Laboratory, Lemont, IL 60439, USA*
[4] *Univ. Grenoble Alpes, CEA Grenoble, IRIG, MEM, NRX, 17 rue des Martyrs, 38000 Grenoble, France*
[5] *ESRF - The European Synchrotron, 71 Avenue des Martyrs, Grenoble 38000, France*
[6] *Present address: ANAXAM, Park Innovaare, Parkstrasse 1, 5234 Villigen, Switzerland*
[7] *Aix Marseille Université, CNRS, Université de Toulon, IM2NP UMR 7334, 13397 Marseille, France*
[8] *Present address: Université Claude Bernard Lyon 1, CNRS/IN2P3, IP2I Lyon, UMR 5822, 69622, Villeurbanne, France*
[9] *Université Claude Bernard Lyon 1, CNRS, Institut Lumière Matière, F-69622 Villeurbanne, France*
[10] *Univ. Grenoble Alpes, CEA Grenoble, IRIG, MEM, NRS, 17 rue des Martyrs, 38000 Grenoble, France*
[11] *CNRS, INSA Lyon, Université Claude Bernard Lyon 1, MATEIS, UMR 5510, 69621 Villeurbanne, France*

## TEM LAMELLA PREPARATION

The NW studied by SXDM was extracted into a TEM lamella by focused ion beam milling. Top and side views of the TEM lamella in preparation in the FIB instrument are shown in Supporting Fig. S1.

## LATTICE MISMATCH WZ-ZB

The lattice mismatch between ZB and WZ phases was estimated though a comparison of the inter-reticular distances $d_{hkl}$ of the different structures and orientations. Eq. E1 was used to estimate the inter-reticular distance in the ZB structure, while Eq. E2 was used for the WZ structure.

$$\mathrm{d}_{\mathrm{hkl}}^{\mathrm{ZB}} = \frac{\mathrm{a}_{\mathrm{ZB}}}{\sqrt{\mathrm{h}^2+\mathrm{k}^2+\mathrm{l}^2}} \quad \text{(E1)}$$

$$\mathrm{d}_{\mathrm{hkl}}^{\mathrm{WZ}} = \frac{1}{\sqrt{\frac{4}{3\mathrm{a}_{\mathrm{WZ}}^2}(\mathrm{h}^2+\mathrm{hk}+\mathrm{k}^2)+\frac{\mathrm{l}^2}{\mathrm{c}_{\mathrm{WZ}}^2}}} \quad \text{(E2)}$$

The experimental lattice parameters of ZB and WZ GaAs were taken as $a_{ZB}$ = 5.6533 Å [1], and $a_{WZ}$ = 3.9845 Å and $c_{WZ}$ = 6.5701 Å [2], respectively. The inter-reticular distances along the growth axis were calculated as $d_{111}$ = 3.2639 Å and $d_{0002}$ = 3.2850 Å for the ZB and WZ structures, respectively, while $d_{2\bar{2}0}$ = 1.9987 Å and $d_{11\bar{2}0}$ = 1.9922 Å were calculated as inter-reticular distances perpendicular to the growth axis, for the ZB and WZ structures, respectively.

The lattice mismatch was determined using equation Eq. E3:

$$\text{lattice mismatch} = \frac{\mathrm{d}^{\mathrm{ZB}}-\mathrm{d}^{\mathrm{WZ}}}{\mathrm{d}^{\mathrm{ZB}}} \times 100 \quad \text{(E3)}$$

leading to ∼-0.6% along the growth axis (ZB[111]) and ∼0.3% perpendicularly to the growth direction. Thus, significant strain can be induced at the interface between ZB and WZ GaAs to accommodate the difference of inter-reticular distance between both polymorphs [3].

* These authors contributed equally to this work

† jose.penuelas@ec-lyon.fr
‡ matthieu.bugnet@insa-lyon.fr

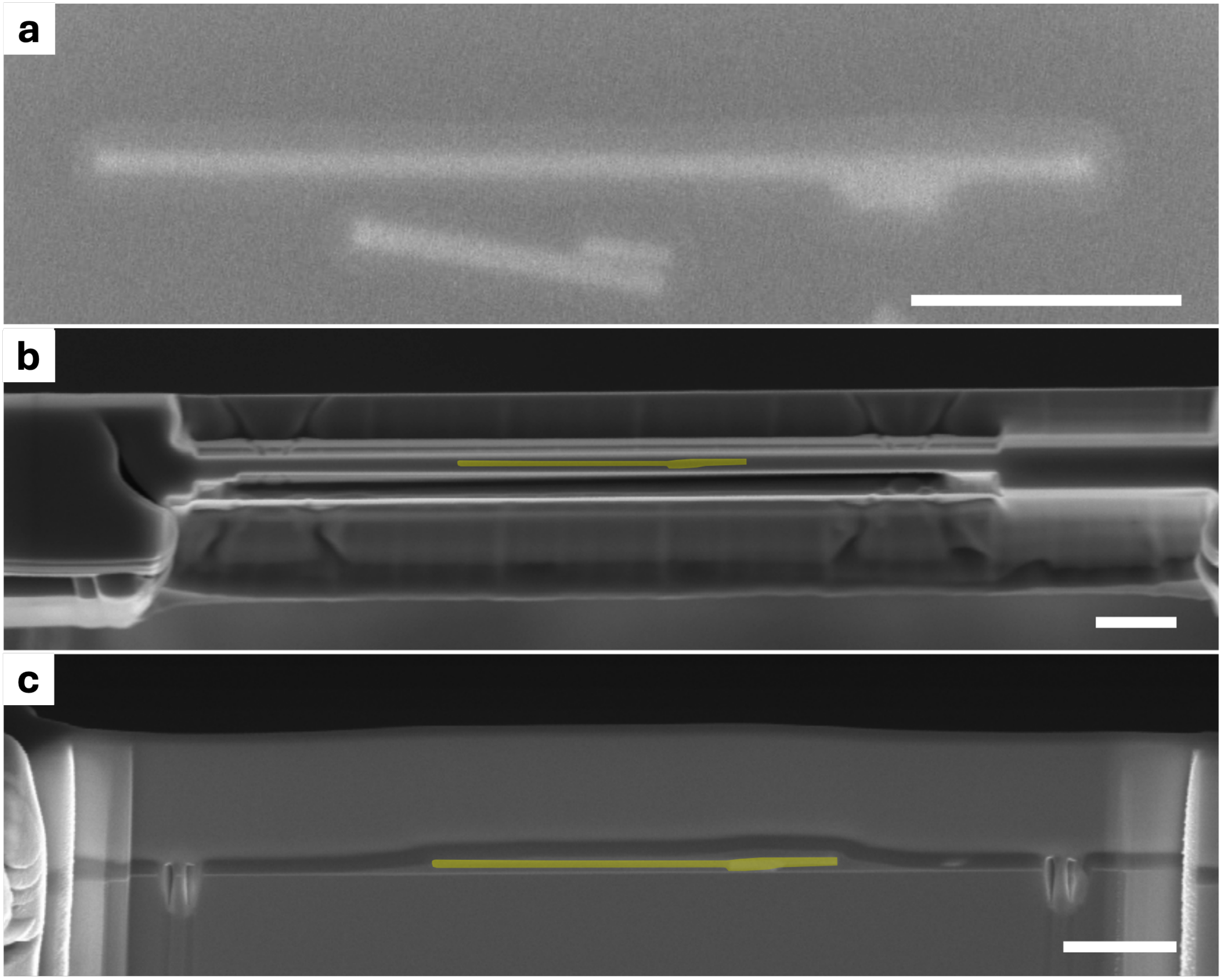


FIG. S1. **Focused ion beam preparation of the nanowire.** a) SEM image of the NW laying on the Si support. b) Top view and c) side view SEM images of the FIB lamella containing the NW investigated on the beamline. Scale bars are 1 µm.

## LINE PROFILES FROM 2D MAPS

The profiles of twist angle $\varphi$ and bend angle $\theta$ shown in Fig. 4 were background subtracted, as illustrated in Supporting Fig. S2. The background is adjusted as best as possible in each case to reflect the shape of the raw profile in the areas of interest along the NW.

## PHASE DISTRIBUTION

Normalizing the phase distribution to 100% for the sum of all three phases, as shown in Supporting Fig. S3, shows a clear correlation with $d$-spacing, twist and bend angles, similar to Fig. 4. The phase distribution shown in Fig. 4 is more representative of the standard deviation of the sum of all phases.

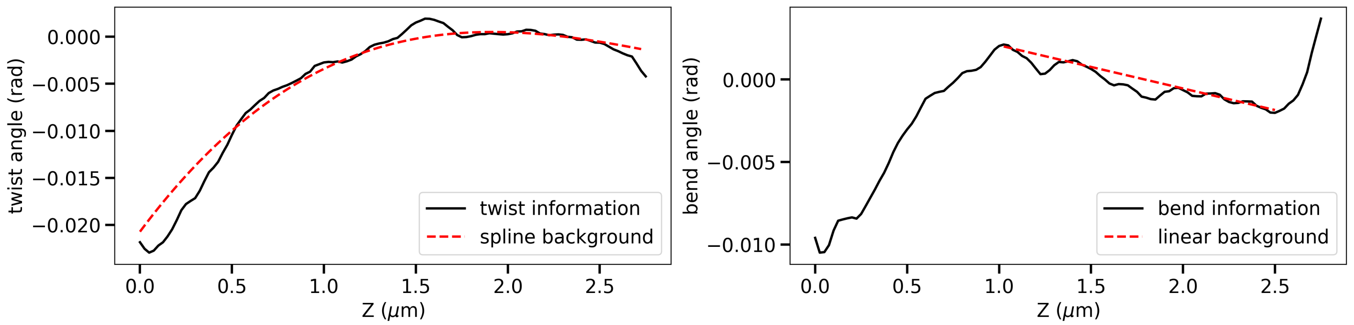


FIG. S2. **Background extraction for twist and bend angles $\varphi$ and $\theta$.** A spline function is fitted for the twist, while a linear background is used for the bend angle.

## LINEAR ELASTICITY MODEL

To rationalize the findings about the $d$-spacing modulations in the NW and their correlation with the crystallographic structures, the deformation of the lattice parameter along the NW was computed from numerical simulations, using a linear elasticity model.

---

The numerical simulation is based on a continuum linear elasticity model, which accounts for the anisotropy of the elastic energy densities of the ZB (cubic) and WZ (hexagonal) phases. The reference configuration is defined by the experimental data for the lattice parameters $a$ (for ZB) and $(a, c)$ (for the WZ phase).

Thus,

$$\psi_{\mathrm{ZB}} = \frac{1}{2}(\mathbb{C}^{\mathrm{ZB}}[\varepsilon]) : \varepsilon, \qquad \psi_{\mathrm{WZ}} = \frac{1}{2}(\mathbb{C}^{\mathrm{WZ}}[\varepsilon - \varepsilon^0]) : (\varepsilon - \varepsilon^0). \tag{E4}$$

where, with respect to the orthonormal frame in the $([11\bar{2}], [\bar{1}10], [111])$ directions, the expressions for $\mathbb{C}^{\mathrm{ZB}}$ and $\mathbb{C}^{\mathrm{WZ}}$ are

$$\mathbb{C}^{\mathrm{ZB}} = \begin{pmatrix} C'_{11} & C'_{12} & C'_{13} & 0 & C'_{15} & 0 \\ & C'_{22} & C'_{23} & 0 & C'_{25} & 0 \\ & & C'_{33} & 0 & 0 & 0 \\ & & & C'_{44} & 0 & C'_{46} \\ & sym. & & & C'_{55} & 0 \\ & & & & & C'_{66} \end{pmatrix}, \qquad \mathbb{C}^{\mathrm{WZ}} = \begin{pmatrix} C'_{11} & C'_{12} & C'_{13} & 0 & 0 & 0 \\ & C'_{22} & C'_{23} & 0 & 0 & 0 \\ & & C'_{33} & 0 & 0 & 0 \\ & & & C'_{44} & 0 & 0 \\ & sym. & & & C'_{55} & 0 \\ & & & & & C'_{66} \end{pmatrix}, \tag{E5}$$

where

$$C'_{11} = C'_{22} = \frac{1}{2}(C_{11} + C_{12} + 2C_{44}), \quad C'_{12} = \frac{1}{6}(C_{11} + 5C_{12} - 2C_{44}), \quad C'_{13} = C'_{23} = \frac{1}{3}(C_{11} + 2C_{12} - 2C_{44}),$$

$$C'_{33} = \frac{1}{3}(C_{11} + 2C_{12} + 4C_{44}), \quad C'_{44} = C'_{55} = \frac{1}{3}(C_{11} - C_{12} + C_{44}), \quad C'_{66} = \frac{1}{6}(C_{11} - C_{12} + 4C_{44}), \tag{E6}$$

$$-C'_{15} = C'_{25} = C'_{46} = \frac{1}{3\sqrt{2}}(C_{11} - C_{12} - 2C_{44}),$$

While for the WZ phase the $([11\bar{2}], [\bar{1}10], [111])$ frame is that of the hexagonal symmetry axes, this is not the case for the ZB phase. Moreover, the ZB and the WZ phases are in contact along a surface whose normal is $[111]_c = [0001]_h$ so that the misfit strain in Eq. (E4) is defined by $\varepsilon^0 = diag(\varepsilon_t, \varepsilon_t, \varepsilon_l)$, where the transversal and longitudinal strains are defined as $\varepsilon_t = (a^{WZ}_{\mathrm{exp}} - a^{WZ}_{\mathrm{id}})/a^{WZ}_{\mathrm{id}}$ and $\varepsilon_l = (c^{WZ}_{\mathrm{exp}} - c^{WZ}_{\mathrm{id}})/c^{WZ}_{\mathrm{id}}$, respectively (id refers to *ideal* values). The

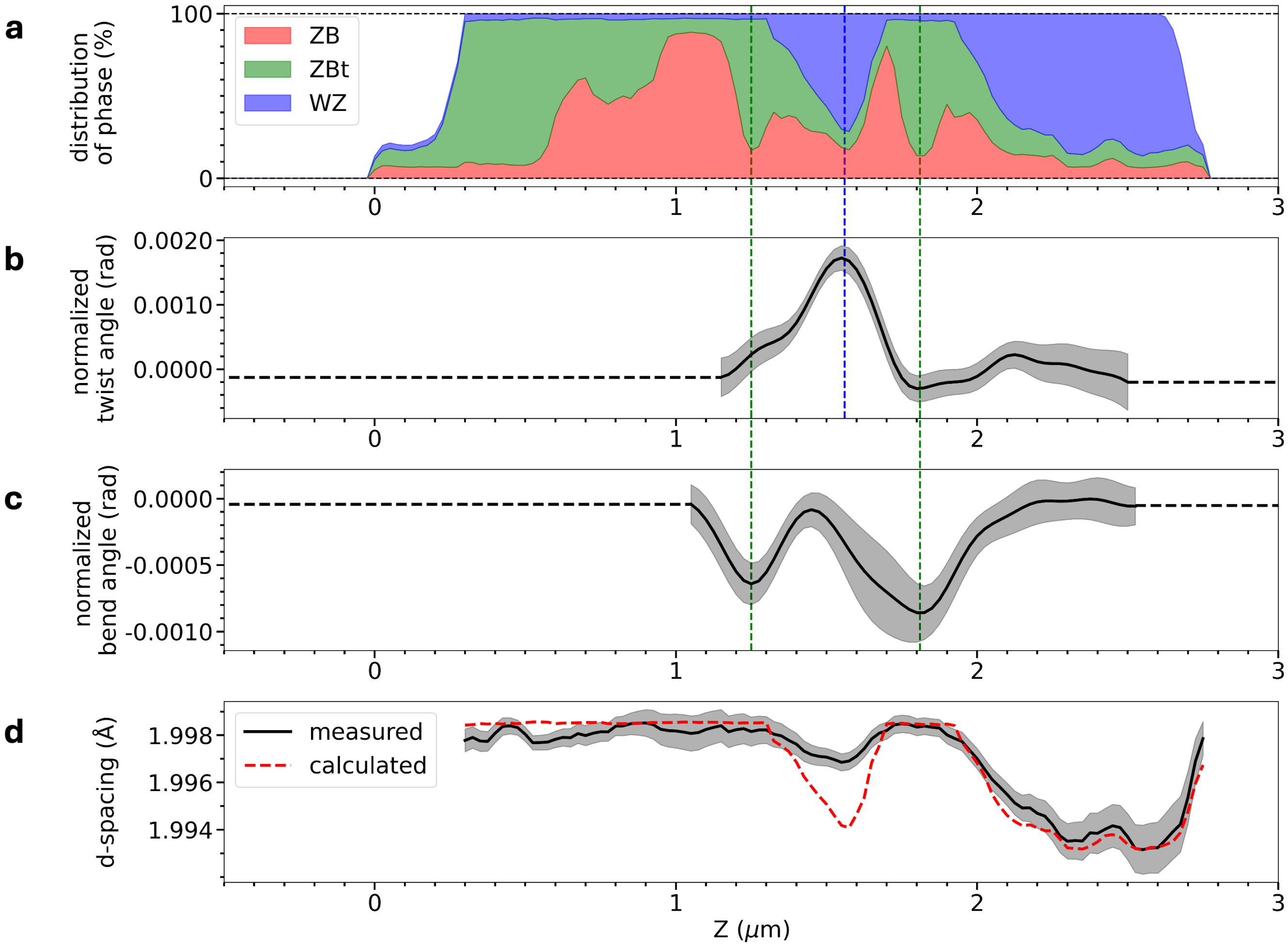


FIG. S3. **Correlation of crystal structure with deformation mechanisms.** Same as Fig. 4, with a forced sum of phase distribution to 100% in (a). a) Normalized intensity of ZB(11$\bar{1}$), ZB$_t$($\bar{1}\bar{1}$3), and WZ(10$\bar{1}$0) diffraction maps shown in Fig. 3, as a function of position along the NW. The intensity of each phase is proportional to its area under the curve. b) Evolution of the twist angle along the NW length, c) bend angle, and d) $d$-spacing. b-d) are line profiles of the 2D-maps shown in Fig. 3, see Methods for details.

experimental values for the lattice parameters in ZB and WZ phases are taken from references 1 and 2, respectively. With this particular choice of the reference configuration both elastic energy minima of pure phases vanish at the experimental values of their respective lattice parameters.

In a NW that contains a sequence of alternate phases, the fact the $a^{\mathrm{WZ}}_{\mathrm{exp}}$ and $c^{\mathrm{WZ}}_{\mathrm{exp}}$ are not equal to their ideal values (i.e. $a^{\mathrm{WZ_{id}}} = a^{\mathrm{ZB_{id}}}/\sqrt{2}$ and $c^{\mathrm{WZ_{id}}} = 2a^{\mathrm{ZB_{id}}}/\sqrt{3}$) induces strains and stress. In order to compare the numerical results with the measured data, the elongation of the $d_{hkl}$ intereticular distance is finally computed through $(\varepsilon n_{hkl}) \cdot n_{hkl}$ and the obtained scalar field is averaged over a volume equal to that of the size of the diffraction spot. While the numerical values for the $C_{11}$, $C_{12}$ and $C_{66}$ in the cubic (ZB) phase are known[4], for the elastic constants of the WZ phase the theoretical estimates given in reference 5 were used.

---

The DF-TEM image shown in Fig. 2a indicates that the long WZ segment at the top of the NW, between Z ~2.3 μm and 2.75 μm contains stacking faults, visible as dark thin lines across the diameter of the NW (Supporting Fig. S4(a)). The top ~450 nm of the NW of the DF-TEM WZ image obtained by selecting the WZ(10$\bar{1}$0) reflection, was segmented to identify the phase location. The phase mapping obtained from this procedure is illustrated in

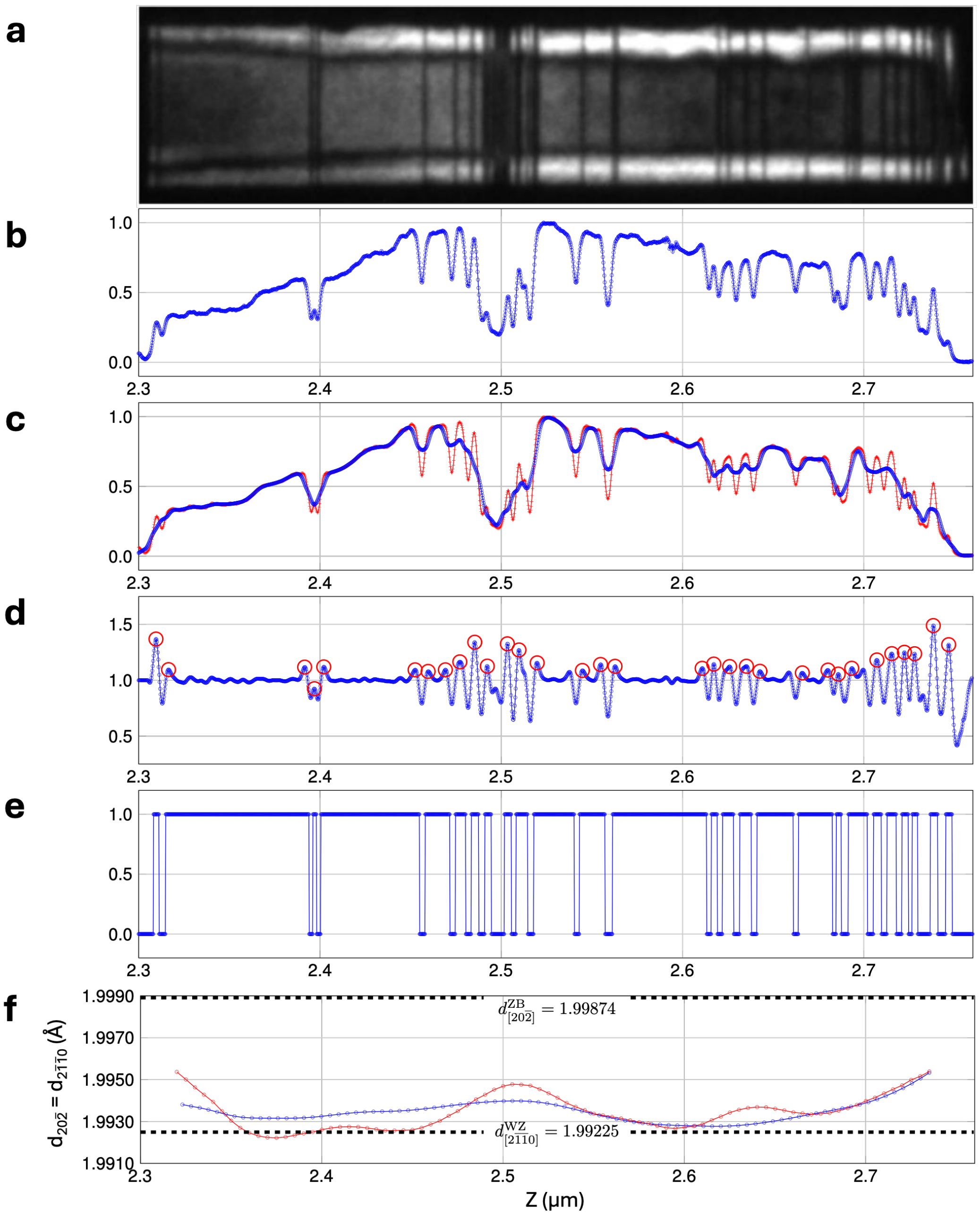


FIG. S4. **Linear elasticity model.** a) DF-TEM image along [$10\bar{1}0$] of the main WZ segment near the head of the NW in the range Z ∼2.3-2.75 µm of Fig. 2. b) Raw intensity summed over the whole width of the nanowire and normalized. c) Denoised intensity (red) and adaptative average signal (blue) obtained after application of a Savitzky-Golay filter. d) Corrected intensity with detected maxima. e) WZ phase detection. A value equal to 1 corresponds to the presence of hexagonal WZ while a value equal 0 designates the presence of a cubic ZB structure. f) Inter-reticular distance ZB $d_{(20\bar{2})}$/ WZ $d_{(2\bar{1}\bar{1}0)}$ measured (blue line) and calculated (red line) for an average NW diameter of 50 nm. Top and bottom dashed lines represent the theoretical values of the ZB $d_{(20\bar{2})}$ and WZ $d_{(2\bar{1}\bar{1}0)}$ inter-reticular distances, respectively.

Supporting Fig. S4(a-e), and was used to calculate the variation of *d*-spacing (Supporting Fig. S4f, also shown in Fig. 5b) along the NW axis using Eqs. E1 and E2 for the ZB and WZ structure, respectively.

For this purpose, the lattice parameters $a$ and $c$ were approximated to their theoretical values in the pure-phase segments and for the portions away from the phase transitions. However, due to the difference of lattice parameters between ZB and WZ, lattice strain is expected close to the areas where stacking faults and structural changes occur, thus slightly modifying the values of the lattice parameters to accommodate these variations.

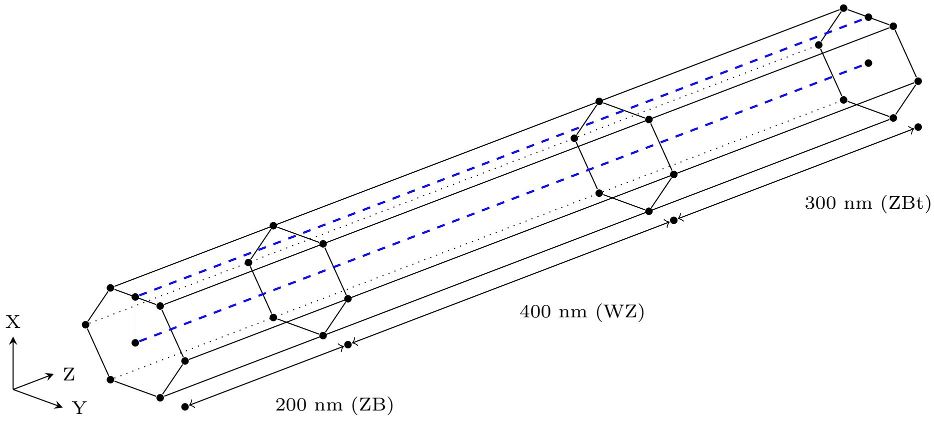


FIG. S5. The geometry of the simulated NW segment: a sequence of 900 nm long containing three segments in the (ZB,WZ,ZBt) crystal phases respectively. The {X,Y,Z} frame is that of the $\{[\bar{1}10], [\bar{1}\bar{1}2], [111]_{ZB} = [0001]_{WZ}\}$ crystallographic directions. We approximate the twist angle as a function of the axial coordinate by $\varphi$(Z) = ($v$(h,0,Z)-$v$(0,0,Z))/($\frac{R\sqrt{(3)}}{2}$) where $v$ is the Y-component of the displacement.

## IMAGE PROCESSING FOR LINEAR ELASTICITY MODEL

The DF-TEM image was converted into a numerical matrix, representing the intensity of each pixel of the image. Since the phase transition only occurs along the long axis of the NW, an intensity profile was calculated by summing the intensity along the width of the NW (Supporting Fig. S4b). The intensity profile obtained was then processed using a Butterworth low-pass filter of order 2, using a wavelength $\lambda_c = 1$ nm. Indeed, the variations smaller than $\lambda_c$ are mainly related to the noise of the measurements and can be neglected. Two different procedures were developed to detect the intensity peaks in the profiles. On the one hand, if the intensity profile only presents equivalent height peaks, a saturation of the signal was used to diminish its dynamic range and reveal the variations of small amplitude. Then, an algorithm identified local maxima and determined their location. The width of each identified peak was determined using the appropriate algorithm. On the other hand, if the signal presents large variation in the peak intensities, a problem occurs in the detection of high intensity variations. To overcome this problem, an adaptive average of the signal is calculated using a Savitzky-Golay filter (Supporting Fig. S4c). Indeed, this filter consists in the calculation of the central point of a sliding window using polynomial interpolation. In our case, a 31 pixel-wide window, corresponding to about 7 nm, was used with a first order polynomial interpolator. The filtered signal is divided by the obtained adaptive average, and maxima are determined (Supporting Fig. S4d). Finally, the algorithm returns tables with values equal to 1 when the phase is detected in the DF-TEM image, and 0 elsewhere (Supporting Fig. S4e).

## FINITE ELEMENT MODELING OF THE TWIST

A recent investigation [6] of one-side stressed GaAs/InAlAs (core-shell) NWs shows that the the coupling between twist and bending may occur in the ZB phase in (vertical) non-symmetric geometries. As in our situation the NW are deposited on a support, we can reasonably assume that the interaction of one of the ZB $[\bar{1}10]$ facets with the support provides elastic energy for the twist.

In order to check this assumption, we performed a 3D numerical simulation of a 900 nm long segment which, in the axial direction, contains alternate ZB/WZ/ZBt phases over $(200, 400, 300)$ nm respectively (see Fig. S5). It represents a rough approximation on the segment located between 1 $\mu$m and 1.9 $\mu$m in Fig. 4. In contrast with assumptions in [6], we limit our study to linear strain measure $\varepsilon = \frac{1}{2}\left(\nabla u + (\nabla u)^T\right)$ and linear stress-strain relation. The interaction with the support was modeled as an (idealized) prestrain along the $[\bar{1}10]$ facet which is in contact with the support (see Fig. S5). The {X,Y,Z} frame for the computation is the $\{[\bar{1}10], [\bar{1}\bar{1}2], [111]\}$-frame in the ZB and ZB$_t$ parts while the [111]-ZB direction is the [0001] direction in the WZ phase.

The axial prestrain induces bending around the X-axis but does not induce twist. In contrast, the lateral $\varepsilon^0_{YY}$ prestrain induces both bending and twist. The twist angle along the vertical section of the NW was obtained by computing the deviation from the vertical direction for segments starting at the NW axis and ending on the upper facet. We notice that in a pure bending deformation (along the X-axis), these segments remain vertical, so that, if

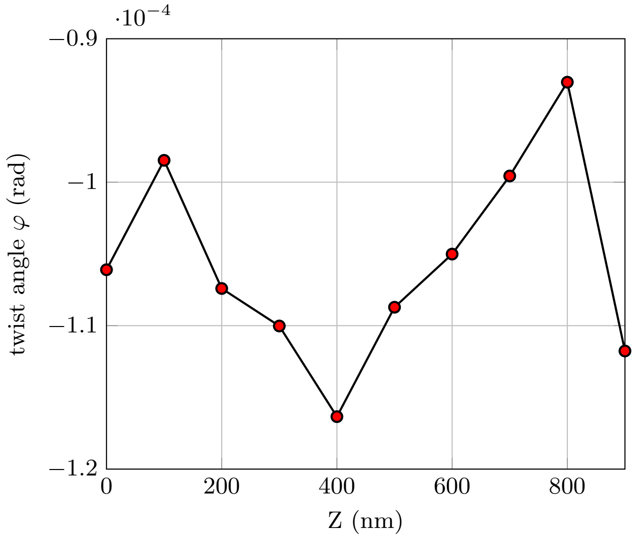


FIG. S6. Values of the twist angle of the vertical section defined by the two blue lines in Fig. S5.

$v$(X,Y,Z) denotes the Y-component of the displacement field, the twist angle along the NW axis can be computed as

$$\frac{1}{h}(v(h,0,Z) - v(0,0,Z)) \tag{E7}$$

where $h = \frac{R\sqrt{(3)}}{2}$ is the distance between the NW axis and the $[\bar{1}10]$ facet and $R$ denotes the NW radius.

The value of the twist angle as a function of the axial coordinate is illustrated in Fig. S6.

[1] S. Saib and N. Bouarissa, High-pressure band parameters for gaas: first principles calculations, Solid-State Electronics **50**, 763 (2006).

[2] D. Jacobsson, F. Yang, K. Hillerich, F. Lenrick, S. Lehmann, D. Kriegner, J. Stangl, L. R. Wallenberg, K. A. Dick, and J. Johansson, Phase transformation in radially merged wurtzite gaas nanowires, Crystal Growth & Design **15**, 4795 (2015).

[3] F. Glas, Strain in nanowires and nanowire heterostructures, in *Semiconductors and Semimetals*, Vol. 93 (Elsevier, 2015) pp. 79–123.

[4] P. Pluengphon, T. Bovornratanaraks, S. Vannarat, and U. Pinsook, Structural and mechanical properties of gaas under pressure up to 200 gpa, Solid State Communications **195**, 26 (2014).

[5] R. M. Martin, Relation between elastic tensors of wurtzite and zinc-blende structure materials, Physical Review B **6**, 4546 (1972).

[6] Y. Hadjimichael, O. Brandt, C. Merdon, C. Manganelli, and P. Farrell, Strain distribution in zincblende and wurtzite gaas nanowires bent by a one-sided (in, al) as stressor shell: consequences for torsion, chirality, and piezoelectricity, arXiv preprint arXiv:2501.09175 (2025).
